# Photobiomodulation Attenuated Cognitive Dysfunction and Neuroinflammation in a Prenatal Valproic Acid-Induced Autism Spectrum Disorder Mouse Model

**DOI:** 10.3390/ijms232416099

**Published:** 2022-12-17

**Authors:** Ui-Jin Kim, Namgue Hong, Jin-Chul Ahn

**Affiliations:** 1 Department of Medical Laser, Graduate School, College of Medicine, Dankook University, Cheonan 31116, Republic of Korea; 2 Medical Laser Research Center, College of Medicine, Dankook University, Cheonan 31116, Republic of Korea; 3 Department of Biomedical Science, College of Medicine, Dankook University, Cheonan 31116, Republic of Korea

**Keywords:** autism spectrum disorder, neuroinflammation, photobiomodulation, valproic acid, cognitive function

## Abstract

Autism spectrum disorder (ASD) is a neurodevelopmental condition characterized by social communication and interaction disorders, as well as repetitive and restrictive behaviors. To date, no effective treatment strategies have been identified. However, photobiomodulation (PBM) is emerging as a promising treatment for neurological and neuropsychiatric disorders. We used mice exposed to valproic acid (VPA) as a model of ASD and found that pathological behavioral and histological changes that may have been induced by VPA were attenuated by PBM treatment. Pregnant mice that had been exposed to VPA were treated with PBM three times. Thereafter, we evaluated the offspring for developmental disorders, motor function, hyperactivity, repetitive behaviors, and cognitive impairment. PBM attenuated many of the pathological behaviors observed in the VPA-induced ASD mouse model. In addition, pathophysiological analyses confirmed that the increase in activated microglia and astrocytes observed in the VPA-induced ASD mouse model was attenuated by PBM treatment. This suggests that PBM can counteract the behavioral changes caused by neuroinflammation in ASD. Therefore, our data show that PBM has therapeutic potential and may reduce the prevalence of neurodevelopmental disorders such as ASD.

## 1. Introduction

Autism spectrum disorder (ASD) is a neurodevelopmental disorder characterized by impaired social communication, as well as restrictive and repetitive behaviors [1]. ASD is also closely linked with neurodegenerative diseases such as schizophrenia, Alzheimer’s disease, Parkinson’s disease, and dementia [2,3]. A report in 2020 from the Centers for Disease Control and Prevention estimated that 1 in 44 children had been diagnosed with ASD. Approximately four times as many males as females are diagnosed with ASD [4]. The cause of autism is not clear, but the most widely accepted explanation is that it is a complex neural developmental disorder characterized by abnormalities in the brain network [5]. According to recent studies, one of the most common risk factors for ASD is ongoing neuroinflammation in various brain regions [6,7]. Recent studies have implicated non-genetic factors in the development of ASD, including exposure to antiepileptic drugs, viral infections, autoimmune diseases, pathogenic substances, and valproic acid (VPA) during pregnancy [8].

VPA is an antiepileptic drug also used to treat bipolar disorder [9]. However, VPA is associated with side effects when administered during pregnancy, including an increased risk of congenital malformation and delayed cognitive development of offspring. Some studies have also suggested that treatment with VPA during pregnancy is associated with an increased risk of ASD, attention deficit disorders, hyperactivity, and fetal valproate syndrome [10].

Photobiomodulation (PBM) uses lasers or light-emitting diodes to apply red or near-infrared light to the brain, thereby improving the metabolic capacity of neurons and stimulating anti-inflammatory responses, antioxidants, neurogenesis, and synaptogenesis [11,12,13,14]. Unlike drug treatments, PBM is noninvasive and has few side effects [15]. PBM improved cognitive rigidity, behavior, attention span, and sleep quality in children with ASD [5]. Recent studies have shown that 635 nm wavelength reduces ASD-related hypersensitivity, among other symptoms [16]. However, the therapeutic mechanism by which PBM influences ASD remains unclear.

We investigated the potential neuroprotective and developmental effects of 830 nm wavelength laser in an ASD model. We identified behavioral changes in the ASD model, which differed according to PBM treatment. We investigated the importance of the developmental stage and performed behavioral tests to assess social interactions, motor function, repetitive behavior, and cognitive function. The neuroinflammatory response occurs due to activated microglia and astrocytes in neurological diseases [17]. According to previous studies, activated microglia and astrocytes are increased in ASD [7,18,19]. PBM is attracting attention as an effective treatment method for neuroinflammation [20]. Therefore, it suggests that PBM can effectively control the neuroinflammation that occurs in ASD. To understand the underlying causes of the changes in behavior, we studied brain samples and found that histological changes in the brain, the neuroinflammatory response, correlated with the pathological findings of behavioral tests.

## 2. Results

### 2.1. Effect of Laser Treatment on VPA-Induced Developmental Abnormalities

**Figure 1:**
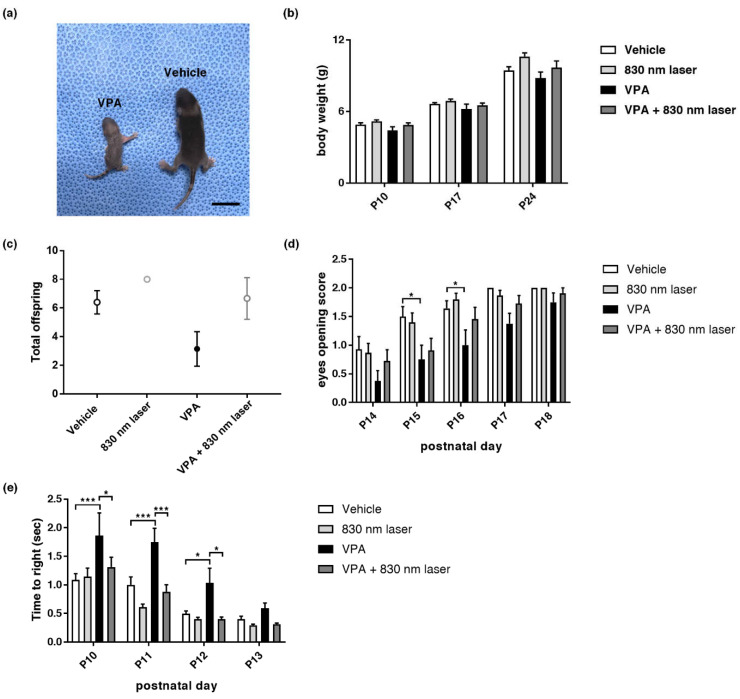
Mice exposed to valproic acid (VPA) in utero exhibited delayed development. (**a**) A pup exposed to VPA was compared to one exposed to the vehicle (postnatal day 9). Scale bar: 1 cm. (**b**) Body weight of mice exposed to VPA (in utero). (**c**) The number of live pups from each pregnancy on postnatal day 10. (**d**) Developmental delays were assessed based on the eye-opening test and (**e**) righting reflex. Eye opening was scored from postnatal days 14 to 18 (score: 0 = closed eyes, 1 = one eye open, 2 = both eyes open). The righting reflex was measured every day from P10 to 13. Data are means ± standard error of the mean (SEM) (vehicle: *n* = 14,830 nm laser: *n* = 15, VPA: *n* = 8, VPA + 830 nm laser: *n* = 11). * *p* < 0.05 and *** *p* < 0.001 compared to the vehicle group. * *p* < 0.05 and *** *p* < 0.001 compared to the VPA group.

Prenatal VPA exposure caused developmental abnormalities in the pups. We found that 830 nm laser treatment attenuated the developmental abnormalities observed in mice exposed to VPA. The effect of 830 nm laser treatment on developmental abnormalities was assessed using body weight measurements, eye opening, and the righting reflex test. Figure 1 (panel 1a,b) shows that VPA decreases body size and weight. Figure 1d shows late eye-opening scores for the VPA group compared to the vehicle group, and improved eye-opening scores for the VPA + 830 nm laser group compared to the VPA group. The total number of live births did not differ significantly between mice exposed to VPA and the vehicle (Figure 1c). The VPA group exhibited late eye-opening, and the differences in eye-opening scores between the vehicle and VPA groups were significant at P14–P17. Eye-opening scores in the VPA + 830 nm laser group improved, with significant differences seen at P14, P16, and P17 (Figure 1d). These data indicate that VPA induced a significant developmental delay in mice, and that the 830 nm laser treatment attenuated this developmental delay. The difference in righting reflex times between the vehicle and VPA groups also highlights the developmental delay in the VPA group, with significant differences seen at P10 and 11. The righting reflex time for the VPA + 830 nm laser group improved, and was significantly different at P11 (Figure 1e). Therefore, VPA induced a significant developmental delay in mice, which the 830 nm laser treatment attenuated.

### 2.2. Laser Treatment Improved Motor Function in Mice Exposed to VPA

**Figure 2:**
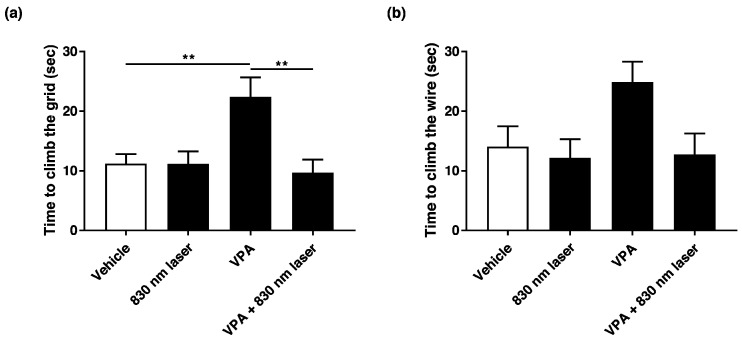
Effect of laser treatment on the motor function of mice exposed to VPA in utero. (**a**) The effect of 830 nm laser treatment on mice exposed to VPA (in utero) was assessed using the negative geotaxis test and (**b**) hanging wire test. Mice were placed on the slope with their heads facing downward. The righting reflex was measured as the time taken for mice to reach the top of the slope. In the hanging wire test, the time elapsed between the holding of the wire with both forelimbs and climbing with the hind limbs was measured. Data are means ± SEM (vehicle: *n* = 14,830 nm laser: *n* = 15, VPA: *n* = 8, VPA + 830 nm laser: *n* = 11). ** *p* < 0.01 compared to the group exposed to VPA.

Prenatal VPA exposure impaired motor function. We found that 830 nm laser treatment improved motor function in mice exposed to VPA. The effect of 830 nm laser treatment on motor function was assessed using a negative geotaxis test (Figure 2a) and a hanging wire test (Figure 2b). The negative geotaxis test revealed a significant delay for the VPA group compared with the vehicle group. However, there was a significant improvement in the VPA + 830 nm laser group compared with the VPA group. These data show that VPA significantly impairs motor function in pups, and that the 830 nm laser treatment improved motor function. The hanging wire test revealed a nonsignificant delay for the VPA group compared with the vehicle group. There was also an improvement in the VPA + 830 nm laser group. Therefore, VPA impaired motor function in the pups, but the 830 nm laser treatment improved motor function.

### 2.3. Laser Treatment Attenuated Social Cognitive Dysfunction in Mice Exposed to VPA

**Figure 3:**
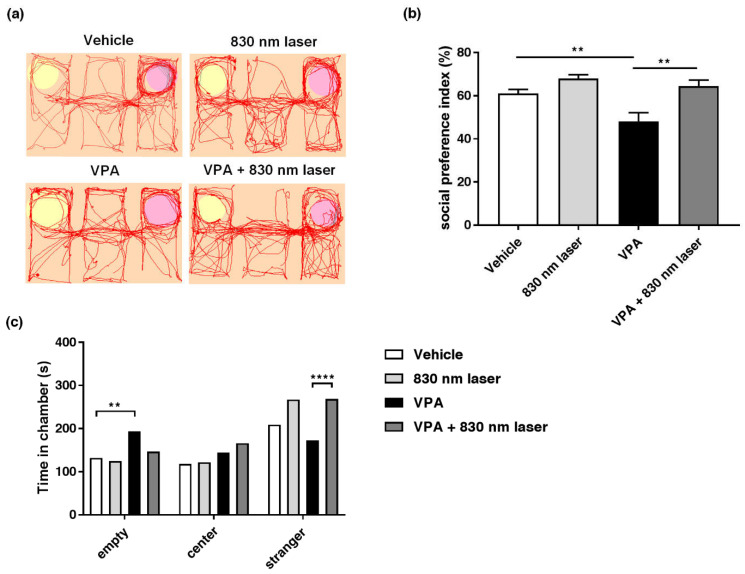
Effect of laser treatment on the social interactions of mice exposed to VPA in utero. (**a**) The effect of 830 nm laser treatment on mice exposed to VPA (in utero) was assessed using the three-chamber test. (**b**) Social preference index (%) = time spent in the stranger chamber divided by the total time spent in all chambers. (**c**) The bar graph indicates that mice spent time in each room, empty, center, and room with a stranger. Data are means ± SEM (vehicle: *n* = 9, 830 nm laser: *n* = 9, VPA: *n* = 6, VPA + 830 nm laser: *n* = 7). ** *p* < 0.01 compared to the vehicle group. **** *p* < 0.0001 compared to the group exposed to VPA.

Impairment of social interaction is one of the core symptoms of ASD, and the three-chamber test is frequently used to investigate social interactions. Mice exposed to VPA exhibited reduced social interaction, i.e., spent little time in the stranger chamber. The 830 nm laser treatment improved social interaction in mice exposed to VPA. The effect of 830 nm laser treatment on social interaction was assessed using the three-chamber test (Figure 3a–c). Mice exposed to VPA exhibited defects in social interaction, spending less time in the stranger than empty chambers compared to vehicle group mice. However, mice in the VPA + 830 nm laser group spent more time in the stranger chamber than mice in the VPA group, indicating improved social interaction.

### 2.4. Laser Treatment Attenuated Impairment in Repetitive Behavior of Mice Exposed to VPA

**Figure 4:**
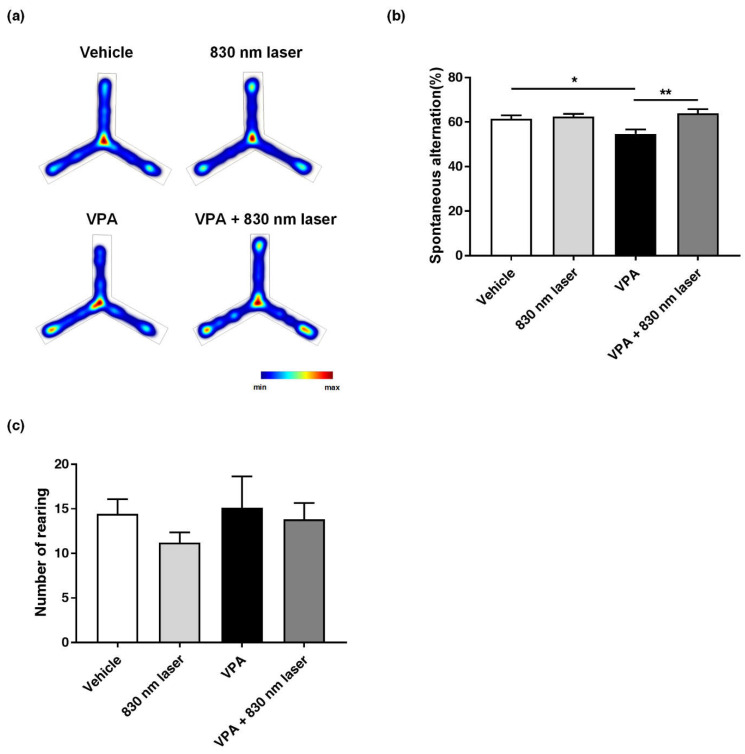
Effect of laser treatment on the repetitive behavior of mice exposed to VPA in utero. The effect of 830 nm laser treatment on mice exposed to VPA (in utero) was assessed using (**a**,**b**) the Y-maze and (**c**) the cylinder rearing test. Spontaneous alternation (%) = total alternations/(total arm entries—2) × 100. Data are means ± SEM (vehicle: *n* = 14,830 nm laser: *n* = 15, VPA: *n* = 8, VPA + 830 nm laser: *n* = 11). * *p* < 0.05 compared to the vehicle group. * *p* < 0.05 and ** *p* < 0.01 compared to the group exposed to VPA.

As with social cognitive dysfunction, impairment in repetitive behavior is one of the core symptoms of ASD. Therefore, we assessed the effect of 830 nm laser treatment on repetitive behavior using the Y-maze (Figure 4a,b) and cylinder test (Figure 4c). The number of forelimb contacts as each mouse reared against the wall of the cylinder was quantified, and there was no significant difference between the VPA and vehicle groups. Although there was an improvement in the VPA + 830 nm laser group, the difference between the VPA + 830 nm laser and VPA groups was not significant. Mice exposed to VPA exhibited impairment in repetitive behavior, and showed a decrease in spontaneous alternation compared to vehicle group mice. Mice in the VPA + 830 nm laser group showed an increase in spontaneous alternation compared to the VPA group mice. These data suggest that the laser treatment attenuated impairment in repetitive behavior of mice exposed to VPA.

### 2.5. Laser Treatment Attenuated Hyperactivity in Mice Exposed to VPA

**Figure 5:**
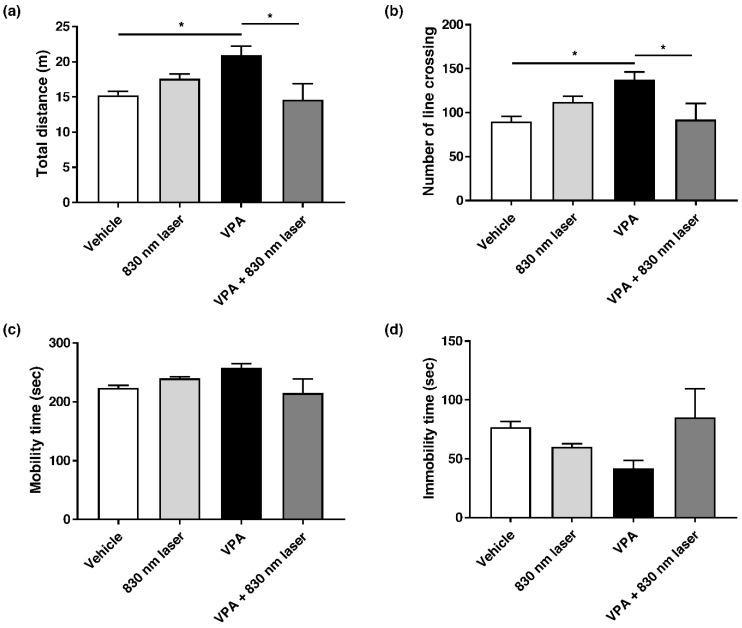
Effect of laser treatment on the hyperactivity of mice exposed to VPA in utero. The effect of 830 nm laser treatment on mice exposed to VPA (in utero) was assessed using (**a**–**d**) the open field test. Data are means ± SEM (vehicle: *n* = 14,830 nm laser: *n* = 15, VPA: *n* = 8, VPA + 830 nm laser: *n* = 11). * *p* < 0.05 compared to the vehicle group. * *p* < 0.05 compared to the group exposed to VPA.

Attention deficit disorder and hyperactivity are common manifestations of ASD [21]. Mice exposed to VPA exhibit hyperactivity. The effect of 830 nm laser treatment on hyperactivity was assessed using the open field test (Figure 5a–d). Compared to mice in the vehicle group, mice exposed to VPA exhibited hyperactivity, reflected in the total distance traveled, number of crossings, and time spent in motion. Mice in the VPA + 830 nm laser group exhibited decreased hyperactivity compared to those in the VPA group.

### 2.6. Laser Treatment Improved Cognitive Function in Mice Exposed to VPA

**Figure 6:**
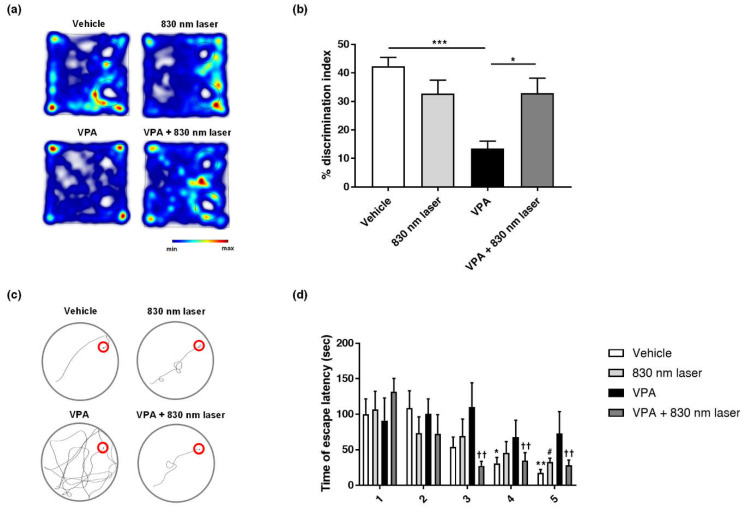
Effect of laser treatment on the cognitive function of mice exposed to VPA in utero. The effect of 830 nm laser treatment on cognitive dysfunction in mice exposed to VPA (in utero) was assessed using (**a**,**b**) the novel object recognition test and (**c**,**d**) Morris water maze. Discrimination index (%) = time spent on the novel object divided by the total time spent exploring both objects. (**b**) The data show significant differences compared to the group exposed to VPA (vehicle: *n* = 9, 830 nm laser: *n* = 9, VPA: *n* = 6, VPA + 830 nm laser: *n* = 7). *** *p* < 0.001 compared to the vehicle group. * *p* < 0.05 compared to the VPA+ 830 nm laser group. (**d**) The data show significant differences compared to the group exposed to VPA (vehicle: *n* = 9, 830 nm laser: *n* = 9, VPA: *n* = 6, VPA + 830 nm laser: *n* = 7). * *p* < 0.05 and ** *p* < 0.01 compared to the vehicle on day 1. # *p* < 0.05 compared to the 830 nm laser group on day 1. †† *p* < 0.01 compared to the VPA + 830 nm laser group on day 1.

VPA impairs the spatial memory of adults and children. The mice exposed to VPA in this study exhibited decreased cognitive function. The effect of 830 nm laser treatment on cognitive function was assessed using the novel object recognition test (Figure 6a,b) and Morris water maze (Figure 6c,d). Mice exposed to VPA exhibited cognitive function deficits, spending less time with novel objects compared to the vehicle group mice. Mice in the VPA + 830 nm laser group exhibited improved cognitive function, spending more time with novel objects compared to VPA group mice. In the Morris water maze, mice exposed to VPA exhibited defective cognitive function, taking longer to reach the platform than vehicle group mice. Mice in the VPA + 830 nm laser group exhibited improved cognitive function, taking less time to reach the platform than VPA group mice.

### 2.7. Laser Treatment Decreased GFAP Expression in the PFC and Hippocampus of Mice Exposed to VPA

**Figure 7:**
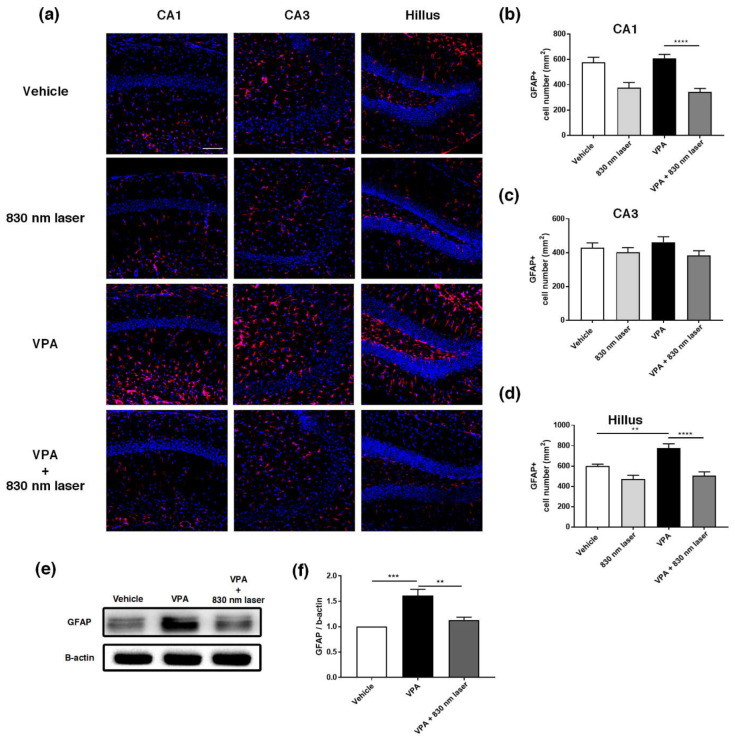
Immunohistochemistry and Western blotting analyses of glial fibrillary acidic protein (GFAP) expression in the hippocampus. (**a**) Image showing GFAP-positive cells (red) in the CA1, CA3, and hilus regions of the hippocampus. (**b**–**d**) Quantification of GFAP-positive cells in the CA1, CA3, and hilus regions of the hippocampus. (**e**) Image of a Western blot. Full-length blots/gels are presented in Figure S1. (**f**) Average relative intensity values for the Western blots. The bar graph summarizes changes in the GFAP-positive cells. Nuclei were stained using 4′,6-diamidino-2-phenylindole (DAPI). Scale bar: 100 µm. Data are means ± SEM. (vehicle: *n* = 3, 830 nm laser: *n* = 3, VPA: *n* = 3, VPA + 830 nm laser: *n* = 3). ** *p* < 0.01 compared to the vehicle group. *** *p* < 0.001 and **** *p* < 0.0001 compared to the group exposed to VPA.

**Figure 8:**
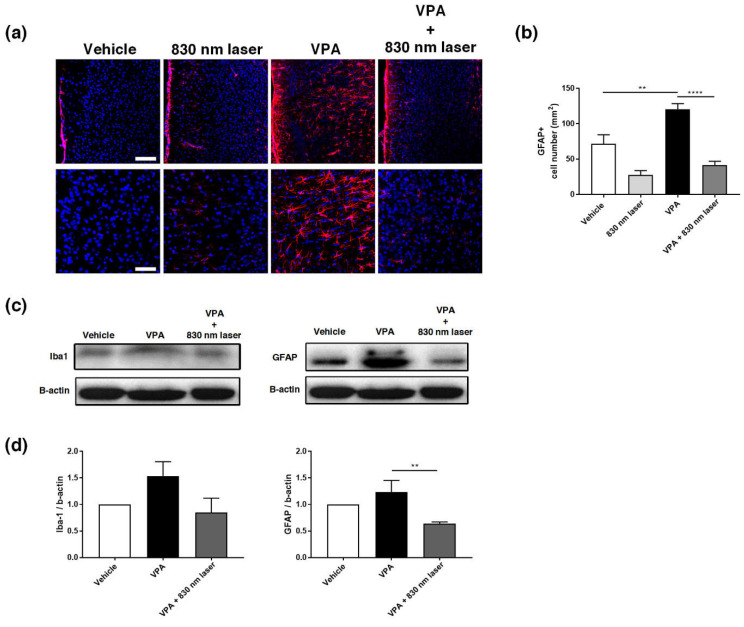
Immunohistochemistry and Western blotting analyses of GFAP expression in the medial prefrontal cortex (mPFC). (**a**) Image showing GFAP-positive cells (red) in the mPFC. (**b**) Quantification of GFAP-positive cells in the mPFC. (**c**) Image of a Western blot. Full-length blots/gels are presented in Figure S2. (**d**) Average relative intensity values for the Western blots. The bar graph summarizes changes in the GFAP-positive cells. Scale bars: 100 and 50 µm. Data are means ± SEM (vehicle: *n* = 3, 830 nm laser: *n* = 3, VPA: *n* = 3, VPA + 830 nm laser: *n* = 3). ** *p* < 0.01 compared to the vehicle group. **** *p* < 0.0001 compared to the group exposed to VPA.

The hippocampus plays an important role in cognitive function [22]. The prelimbic area of the medial PFC (mPFC) is involved in decision-making, cognitive function, attention, and motor activities [23,24]. Because astrocytes are involved in learning and the regulation of long-term memory [25], we performed immunohistochemical analyses of the hippocampus (Figure 7a) and mPFC (Figure 8a) using an antibody for GFAP. Quantitative analyses revealed significant statistical differences in the number of GFAP^+^ astrocytes in the hippocampus and PFC, not only for the vehicle group, but also the VPA + 830 nm laser group compared with the VPA group. The number of GFAP^+^ astrocytes in the CA1, CA3, and hilus regions of the hippocampus was significantly decreased in the VPA + 830 nm laser group compared with the VPA group (Figure 7b–d). The number of GFAP^+^ astrocytes in the mPFC also significantly decreased in the VPA + 830 nm laser group compared with the VPA group (Figure 8b). Western blotting was used to quantify the expression of GFAP (Figure 7e,f and Figure 8c,d). The full-length blots are presented in Figures S1 and S2.

### 2.8. Laser Treatment Decreased Iba1 Expression in the Hippocampus of Mice Exposed to VPA

**Figure 9:**
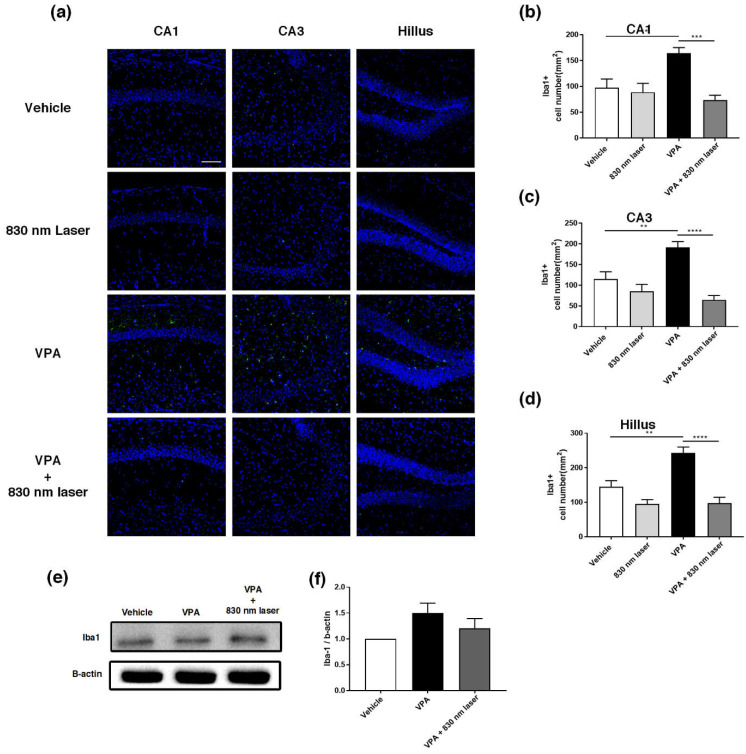
Immunohistochemistry and Western blotting analyses of ionized calcium-binding adapter molecule 1 (Iba1) expression in the hippocampus. (**a**) Image showing Iba1-positive cells (green) in the CA1, CA3, and hilus regions of the hippocampus. (**b**–**d**) Quantification of Iba1-positive cells in the CA1, CA3, and hilus regions of the hippocampus. (**e**) Image of a Western blot. Full-length blots/gels are presented in Figure S1. (**f**) Average relative intensity values for the Western blots. The bar graph summarizes changes in the Iba1-positive cells. Nuclei were stained using DAPI. Scale bar: 100 µm. Data are means ± SEM. (vehicle: *n* = 3, 830 nm laser: *n* = 3, VPA: *n* = 3, VPA + 830 nm laser: *n* = 3). ** *p* < 0.01 compared to the vehicle group. *** *p* < 0.001 and **** *p* < 0.0001 compared to the group exposed to VPA.

Microglia are macrophages activated when the central nervous system is damaged by infection or disease [26]. Increasing evidence indicates that microglial cell activation and dysfunction can have a profound effect on neurodevelopment, leading to neurodevelopmental disorders including autism [27]. In a previous study, RNA sequencing revealed a close association between genes involved in ASD and glial cell activation, and genes involved in immune and inflammatory responses [28]. Because microglial activation is involved in developmental disorders [29], we performed immunohistochemical analyses of the hippocampus (Figure 9a) and mPFC using an antibody for Iba1. Quantitative analyses revealed significant differences in the number of Iba1^+^ microglia cells in the hippocampus, not only for the vehicle group, but also for the VPA + 830 nm laser group compared with the VPA group. The number of Iba1^+^ microglia in the CA1, CA3, and hilus regions of the hippocampus (but not the mPFC) was also significantly decreased in the VPA + 830 nm laser group compared with the VPA group (Figure 9b–d). Western blotting was used to quantify the expression of Iba1 (Figure 9e,f and Figure S1). 

## 3. Discussion

Prenatal exposure of mice to VPA can be used to mimic the pathogenesis of ASD and provide important insight into the morphological and behavioral characteristics of the disease [30]. However, the mechanism by which VPA generates the ASD phenotype remains unclear. In mice, neurogenesis occurs from E12 until the late embryonic developmental stage [31], and exposure to VPA disrupts normal neurogenesis [32]. Intrauterine exposure to VPA may cause ASD symptoms such as developmental abnormalities, social and communication disorders, and restrictive behaviors [33,34,35]. Especially, it was reported that the intraperitoneal injection to maternal of VPA 400-600mg/kg on 12 embryonic days could have a symptom related to autism such as social impairments, cognitive rigidity, and repetitive behaviors [36]. We generated an ASD model by exposing mice to VPA at E12 and confirmed the symptoms of ASD using behavioral tests. The battery of behavior tests was performed in the least stressing and challenging order to avoid potential training and learning effects [37]. Our data suggest that VPA exposure inhibits neurogenesis and triggers the onset of ASD and behavioral disorders.

In this study, we investigated whether PBM treatment during fetal development could attenuate the symptoms of ASD in a mouse model. PBM treatment with an 830 nm laser attenuated the developmental and behavioral abnormalities observed in a VPA-induced ASD mouse model. 

PBM has been confirmed to improve memory decline, amyloid plaques, tau hyperphosphorylation, neurodegeneration, spinal damage, and synapse loss due to the neuroprotective effect of 808nm PBM in Alzheimer’s disease, which shows cognitive deficits among various neurological diseases [38]. In addition to this, NIR shows several neuroprotective effects in animal models with various neurological diseases such as brain stroke and Parkinson’s disease [39,40]. Dysregulation of Neurogenesis is caused by the neuroinflammatory response [41], and PBM can promote neurogenesis by stimulating neural progenitor cells [42,43]. Because 830 nm PBM has the lowest absorption rate of water and hemoglobin in tissue, it has better tissue penetration in the deeper range and can reduce nerve damage [44,45,46]. This was expected to reduce neuroinflammation due to VPA using the 830 nm wavelength. Our results confirmed through GFAP and Iba1 that 830 nm laser-activated astrocytes and microglia were reduced. Therefore, PBM may counteract intrauterine exposure to VPA at E12.5 and prevent the inhibition of neurogenesis.

As well as developmental abnormalities, we also observed various behavioral changes in our VPA-induced ASD mouse model. Reduced motor function is often characteristic of ASD [37]. Using the wire maneuver and negative geotaxis tests, we confirmed that the VPA-induced ASD mouse model exhibited a decrease in motor function that was relieved by PBM. However, we did not observe corresponding neuronal changes in the motor cortex, which is responsible for motor function.

The three-chamber and Y-maze test results demonstrated deficiencies in social interaction, as well as repetitive and restrictive behaviors, in our VPA-induced ASD mouse model, which are all symptoms of ASD. In addition, these symptoms were attenuated by PBM treatment. Our histological studies also revealed corresponding neurological changes in the mPFC region of the brain, which is particularly important for social cognition and behavior [47].

Children with autism often exhibit cognitive deficiencies [48]. Our VPA-induced ASD mouse model exhibited cognitive deficiencies that were attenuated by PBM. Our histological studies also revealed corresponding changes in the hippocampus, which may affect cognitive function [22]. Previous studies have shown that exposure to VPA can alter oxidation status and the expression of proinflammatory genes [49]. Microglia and astrocytes are frequently activated in neurodegenerative diseases [50,51], and have also been found in postmortem brain tissue from patients diagnosed with ASD [7,18,19].

Glial fibrillary acidic protein (GFAP) is a structural marker protein of astrocytes, and ionized calcium-binding adapter molecule 1 (Iba1) is mainly used as an activation marker of microglia [52,53]. In response to CNS damage, the increase in activated glial cells causes cytokine activation, resulting in a gliosis reaction affecting nerve cells, resulting in a neuroinflammatory response [54]. Therefore, we confirmed the activation of astrocytes and microglia through the expression of GFAP and Iba1 in the ASD group exposed to VPA, and demonstrated that neuroinflammation was reduced through the 830 nm laser reducing their expression.

In summary, PBM may decrease neuroinflammation and improve cognitive function in mice exposed to VPA by deactivating microglia and astrocytes. Our results suggest that deficiencies in cognitive function associated with ASD are attenuated when PBM reduces the activity of microglia and astrocytes in the hippocampus. Therefore, this study demon-strates that PBM treatment may benefit patients with ASD and related neurodegenerative diseases.

## 4. Methods and Materials

### 4.1. Animals

C57/BL6 mice were purchased from Daehan Biolink Co., Ltd. (Samseong-myeon, Republic of Korea) and maintained in the Dankook University Laser Research Center (Yongin, Republic of Korea) under a 12 h light/dark cycle on an autonomic nervous system diet. All experiments were conducted in compliance with the NIH Guidelines for the Care and Use of Laboratory Animals and approved by the Institutional Animal Care and Use Committee (IACUC; DKU-20-036) of Dankook University. Both sexes were assayed.

### 4.2. Prenatal VPA Mouse Model for ASD

**Figure 10:**
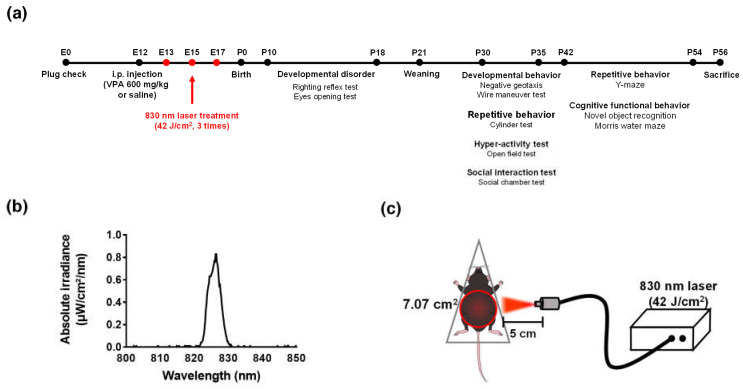
The diode laser. (**a**) Timetable. (**b**) Laser spectrum at a wavelength of 830 nm. (**c**) Schematic diagram showing laser irradiation of the abdomen (diameter: 7.07 cm^2^).

Experiments were performed on 8-week-old adult male and female mice. C57/BL6 mice were housed together overnight to obtain pregnant mice for the prenatal model. After overnight mating, a vaginal plug was applied to pregnant female mice on embryonic day 0 (E0) [55]. On embryonic day 12 (E12), pregnant mice received a single intraperitoneal injection of 600 mg/kg VPA (P4543; Sigma-Aldrich, St. Louis, MO, USA) dissolved in saline. Control female mice were treated with the vehicle (i.e., saline solution) only. Then, prenatal PBM mice were placed in a decapicone (MDC200; Braintree Scientific, Inc., Braintree, MA, USA) and treated three times with a laser (830 nm, 70 mW/cm^2^, 10 min, Figure 10a–c, Table 1) The laser setup was based on previous work [56]. After weaning (P21), the male and female mice in each group were kept in different cages. The number of pups in each group at P10 was as follows: vehicle, *n* = 14; 830 nm laser, *n* = 15; VPA, *n* = 8; and VPA + 830 nm laser, *n* = 11. Both males and females were used in behavioral experiments, but females were not included in the data because they did not show behavioral changes (Figure S3).

### 4.3. Behavioral Tests

The expression of the ASD-like phenotype and effects of PBM treatment were evaluated by behavioral tests performed during the period P8–54 (Figure 10), between 10:00 am and 6:00 pm on the test days. Developmental disorder, SHIRPA, hyperactivity, social interaction, and cognitive behavior tests were completed. Mice were transferred from the housing cage to the experimental chamber 30 min before each test, to habituate to the test environment.

#### 4.3.1. Eye Opening Test

Eye opening was observed once daily from P14 to P18 and scored as follows: 0 = both eyes closed, 1 = one eye open, and 2 = both eyes open [37].

#### 4.3.2. Righting Reflex Test

The righting reflex test measured the pup’s ability to regain its footing from the supine position [57]. At P10–13, the pups were placed on their backs on a flat surface and held in that position for 5 s. The time it took for each pup to return to the prone position after being released was recorded.

#### 4.3.3. Bodyweight

Weight was measured on P10, P17, and P24.

#### 4.3.4. Negative Geotaxis

Negative geotaxis was measured by placing each pup face down along a 45° incline. The time taken to climb after turning 180° was recorded [58]. If the mouse did not climb within 30 s, the maximum time was recorded.

#### 4.3.5. Wire Maneuver Test

A wire maneuver test was used to assess whole-body force [59]. A metal wire was maintained horizontally 20 cm above a thick layer of soft bedding. Each mouse was allowed to grasp the wire by its forepaws. The time taken for each mouse to climb onto the wire was recorded. If the mouse did not raise its hind paws within 3 min, the maximum time was recorded.

#### 4.3.6. Three-Chamber Test

The sociability test involved a rectangular box (60 cm × 40 cm × 20 cm) divided into three chambers with holes that allowed movement between the chambers. Each test was started after 10 min habituation and lasted for 10 min. During the test, a new “stranger” mouse was placed in the holding cage of one chamber. The time spent in each chamber with/without the stranger mouse was measured. All recorded videos were analyzed using EthoVision XT software (version 15.0; Noldus, Wageningen, The Netherlands). The social preference index was calculated as follows: Social preference index (%) = Time in stranger chamber/(Time in empty chambers + Time in stranger chamber) × 100 [60].

#### 4.3.7. Y-Maze

The Y-maze spontaneous alternation test is used to assess repetitive behavior and spatial working memory [61]. All mice were placed in the same arm of the maze and allowed to explore it for 5 min. The maze was cleaned with 70% ethanol after each animal was tested. All recorded videos were analyzed by Ethovision XT software (version 15.0; Noldus, Wageningen, The Netherlands) in terms of the number of alternating entries. Spontaneous alternation was calculated as follows: Spontaneous alternation (%) = Total alternations/(Total arm entries—2) × 100 [33].

#### 4.3.8. Cylinder Test

The cylinder test involved placing each mouse in a transparent cylindrical chamber for 3 min. This test evaluates locomotor asymmetry by measuring the number of forelimb contacts as the mouse rears against the wall of the cylinder [62].

#### 4.3.9. Open Field Test

The open field test was performed as described previously [63] to assess hyperactivity at P35. Test mice were placed in the center of a cube-shaped open field arena (50 cm each side) and allowed to explore freely for 5 min. The arena was cleaned with 70% ethanol after each animal was tested. All recorded videos were analyzed by Ethovision XT software (version 15.0; Noldus, Wageningen, The Netherlands) in terms of the total distance covered and the number of times a line was crossed, as well as the total time spent in motion/stationary.

#### 4.3.10. Novel Object Recognition Test

The novel object recognition test used the same apparatus as the open field test [64]. The test consisted of three phases: habituation, training, and testing. The habituation phase lasted for 5 min; the animals were exposed to the arena and then returned to the home cage while the arena was cleaned. During the training phase, each animal was placed in the arena with two identical objects located in opposite corners. The animal was returned to the home cage for 10 min to ensure that its memory was being tested. During the test phase, a familiar object and unfamiliar object were placed in the arena. Exploratory activity was monitored for 10 min in each test. Preference for the novel object was defined as the ratio of time spent with the novel object compared to that spent with the familiar object. All recorded videos were analyzed by Ethovision XT software (version 15.0; Noldus, Wageningen, The Netherlands) and the time spent sniffing each object was measured. The discrimination index was calculated as follows: Discrimination index (%) = Time spent on novel object/Total time spent exploring both objects [65].

#### 4.3.11. Morris Water Maze

The Morris water maze apparatus was a circular metal box (diameter: 100 cm) with distinct visual cues placed at the quadrant points [66]. White nontoxic paint was applied to hide the platform below the water. The water was maintained at 20–25 °C. Each test lasted 180 s, or until the mouse found the platform. A sequence of five trials was performed over 5 days. All recorded videos were analyzed by Ethovision XT software (version 15.0; Noldus, Wageningen, The Netherlands).

### 4.4. Immunohistochemistry

Mouse brains were fixed in 4% paraformaldehyde overnight at 4 °C, and then dehydrated overnight at 4 °C by incubation in a series of sucrose solutions (10%, 20%, and 30%). Whole brains were then embedded in optimal cutting temperature compound and cryosectioned into 18 mm slices using a cryostat (Leica, Wetzlar, Germany). For immunohistochemical analyses, frozen sections were washed with 0.1 M phosphate-buffered saline (PBS) before permeabilization with 0.5% Triton X-100 for 5 min at room temperature. Sections were blocked with 5% bovine serum albumin (BSA) in 0.3% Triton X-100 in 1× PBS for 1 h 30 min at room temperature. Next, sections were incubated overnight with primary antibodies against the following in 1% BSA and 0.3% Triton X-100: glial fibrillary acidic protein (GFAP; mouse, 1:500; MAB360; Sigma-Aldrich, Burlington, MA, USA) or ionized calcium-binding adapter molecule 1 (Iba1; rabbit, 1:250; PA5-27436; Thermo Fisher, Waltham, MA, USA). Finally, sections were incubated with secondary antibody (Alexa Fluor 488- or 555-conjugated antibody) for 1 h at room temperature before being washed with 1× PBS and mounted in a solution containing 4′,6-diamidino-2-phenylindole (DAPI).

### 4.5. Western Blot Analysis

The hippocampus and prefrontal cortex (PFC) were dissected from male pups in all groups (P56). Tissue was homogenized in ice-cold radio-immune precipitation assay buffer with protease inhibitor, followed by protein isolation and quantification in a detergent-compatible assay. Equal concentrations of protein (30 µg) were loaded onto 15% sodium dodecyl sulfate-polyacrylamide gel electrophoresis gels and then transferred to polyvinylidene difluoride membranes. The membranes were blocked with 5% BSA for 1 h and then incubated overnight at 4 °C with the following primary antibodies: anti-GFAP (1:1000; MAB360; Sigma-Aldrich, Burlington, MA, USA), anti-Iba1 (1:500; PA5-27436; Thermo Fisher, Waltham, MA, USA), or anti-β-actin (1:5000). Then, the membranes were washed with 1× Tris buffered saline with Tween-20 (TBST) buffer (5 min, three times) and incubated with horseradish peroxidase-bound secondary antibody for 1 h. After washing with TBST, Western blot images were acquired using a Chemi Doc system (Bio-Rad, Hercules, CA, USA). Images were analyzed using ImageJ software (version 1.5; National Institutes of Health, Bethesda, MD, USA).

### 4.6. Statistics

Data are reported as means ± standard error of the mean (SEM) and analyses were performed using GraphPad Prism software (ver. 7.0; GraphPad Software, Inc., San Diego, CA, USA). Data were analyzed using one-way analysis of variance (ANOVA) or two-way ANOVA followed by the Bonferroni test. A *p*-value < 0.05 was considered statistically significant.

## Figures and Tables

**Table 1:** Specifications for laser parameters.

Parameter	Value
Wavelength	830 nm
Operating mode	Continuous
Distance	5 cm
Beam shape	Circular
Spot size	Diameter = 7.07 cm^2^
Power density (mW/cm^2^)	70 mW/cm^2^
Exposure duration (min)	10 min
Energy density (J/cm^2^)	42 J/cm^2^
Application technique	Without skin contact on the abdomen
Number and frequency of treatment sessions	3 sessions, once for 2 days

## Data Availability

The data that support the findings of this study are available from the corresponding author upon reasonable request.

## Supplementary Materials

The supporting information can be downloaded at: https://www.mdpi.com/article/10.3390/ijms232416099/s1.
