# Supplementary material for: Photobiomodulation Attenuated Cognitive Dysfunction and Neuroinflammation in a Prenatal Valproic Acid-Induced Autism Spectrum Disorder Mouse Model
Source: Int J Mol Sci. 2022 Dec 17;23(24):16099. doi: 10.3390/ijms232416099 (PMC9785820; doi:10.3390/ijms232416099)
Supplement: Supplementary file 1 [file ijms-23-16099-s001.zip › ijms-2065159-supplementary.pdf]

Supplementary figures

Figure S1: Full-length blots of neuroinflammatory markers in hippocampus. (a) β-actin of GFAP and Iba1, (b) GFAP, (c) Iba1

Figure S2: Full-length blots of neuroinflammatory markers in medial prefrontal cortex. (a) β-actin of GFAP and Iba1, (b) GFAP, (c) Iba1

Figure S3: Results of behavior test into female offspring. (A) Results of body weight (B) Righting reflex test (C) Eyes opening test (D) Negative geotaxis (E) Wire maneuver test (F) Three chamber test (G) Cylinder rearing test (H) Y-maze test (I) Novel object recognition (J) Morris water maze

Supplementary figures

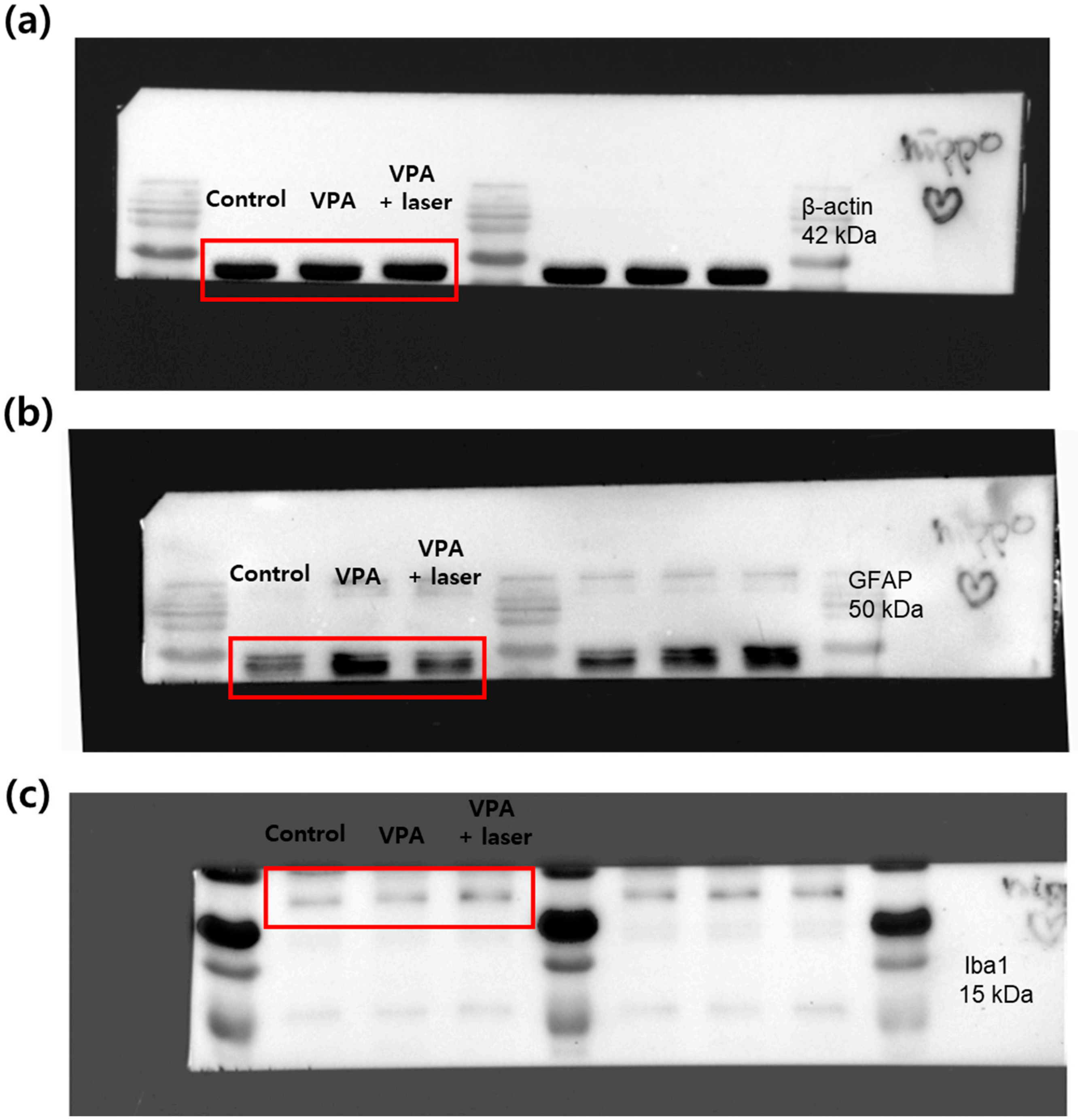

Figure S1. Full-length blots of neuroinflammatory markers in hippocampus. (a) β-actin of GFAP and Iba1, (b) GFAP, (c) Iba1

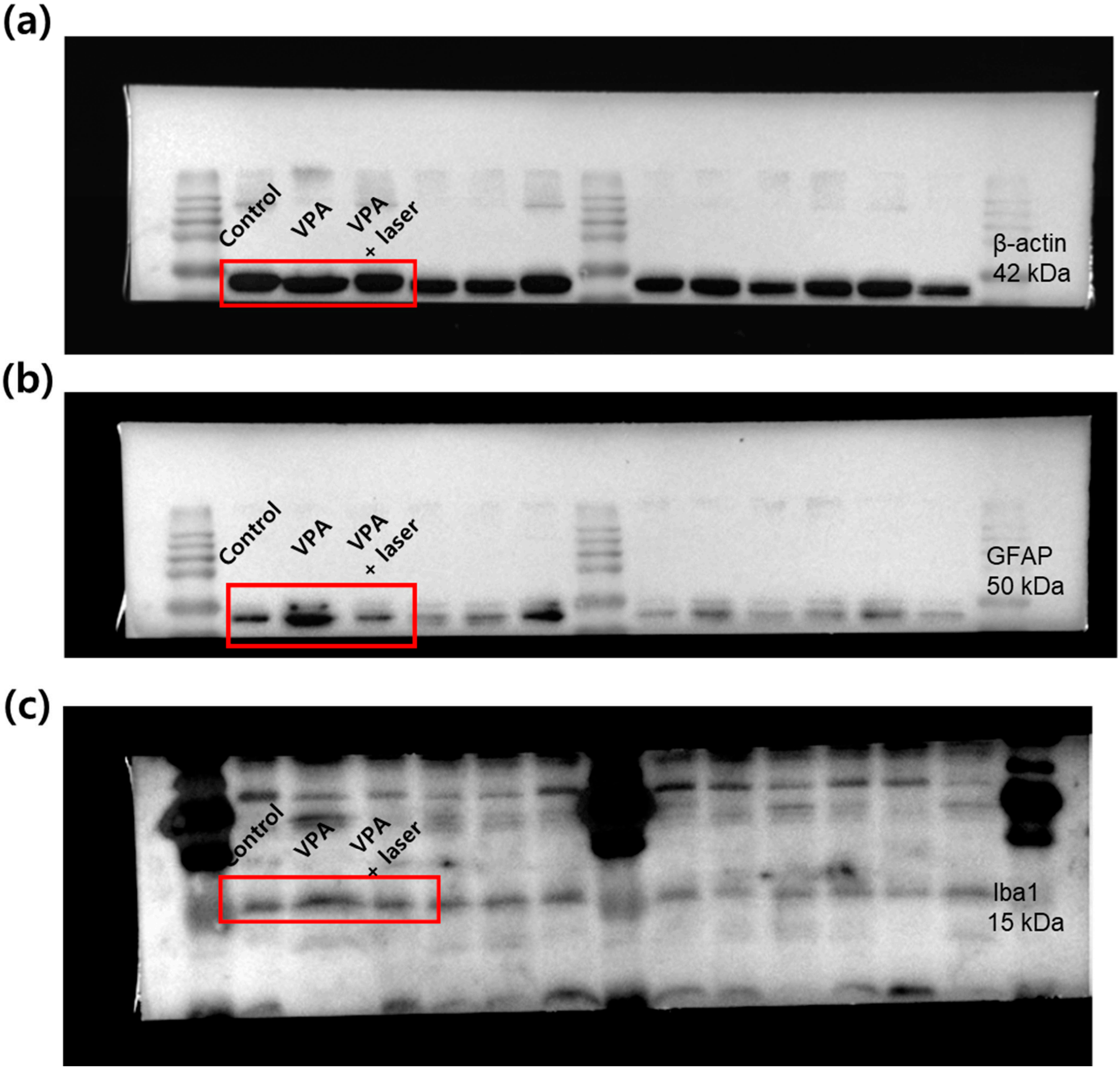

Figure S2. Full-length blots of neuroinflammatory markers in medial prefrontal cortex. (a) β-actin of GFAP and Iba1, (b) GFAP, (c) Iba1

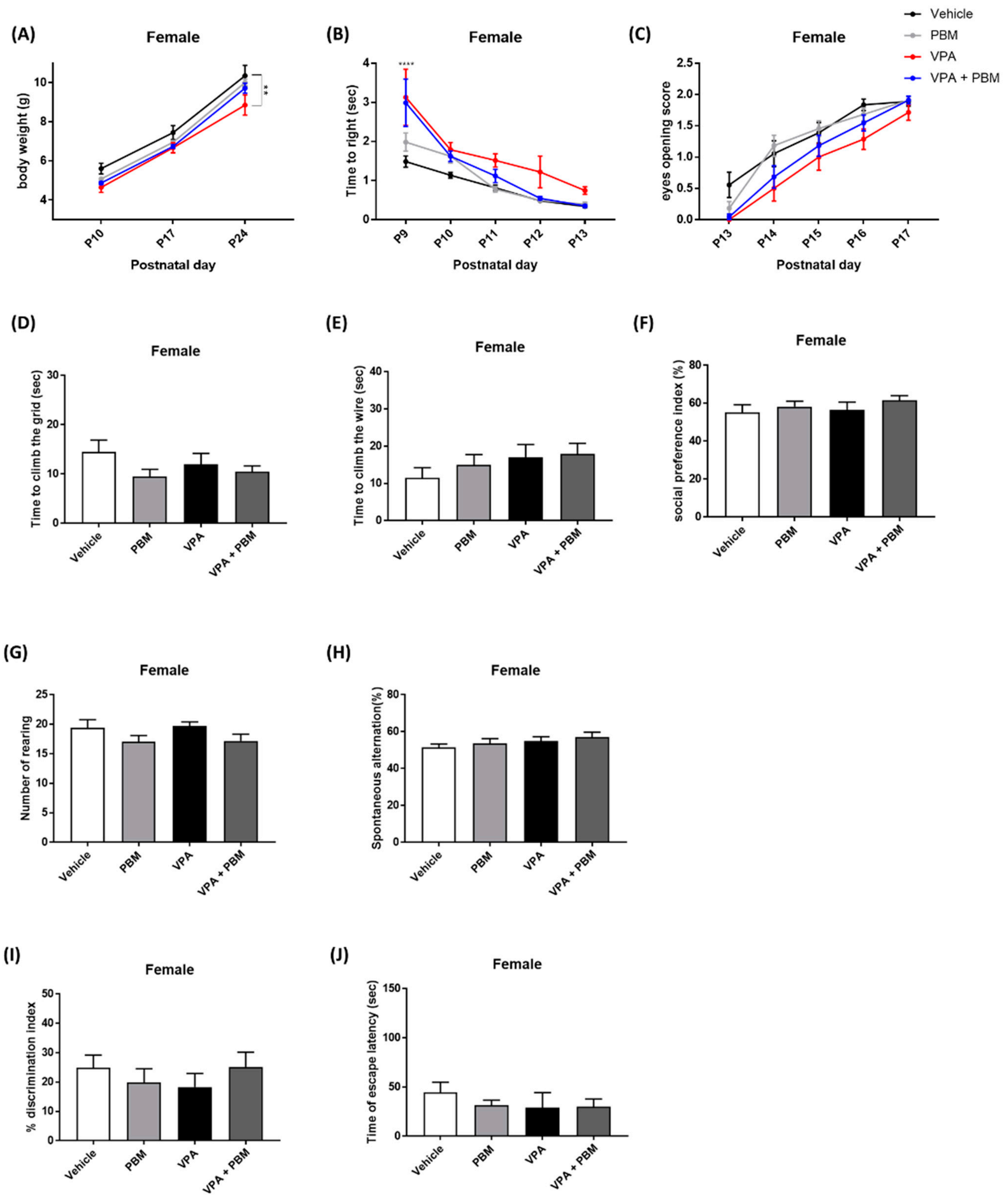

Figure S3. Results of behavior test into female offspring. (A) Results of body weight (B) Righting reflex test (C) Eyes opening test (D) Negative geotaxis (E) Wire maneuver test (F) Three chamber test (G) Cylinder rearing test (H) Y-maze test (I) Novel object recognition (J) Morris water maze
